# Investigation of PRDM10 and PRDM13 Expression in Developing Mouse Embryos by an Optimized PACT-Based Embryo Clearing Method

**DOI:** 10.3390/ijms22062892

**Published:** 2021-03-12

**Authors:** Jiwon Woo, Byung-Ho Jin, Mirae Lee, Eunice Yoojin Lee, Hyung-Seok Moon, Jeong-Yoon Park, Yong-Eun Cho

**Affiliations:** 1 Department of Neurosurgery, The Spine and Spinal Cord Institute, Gangnam Severance Hospital, Yonsei University College of Medicine, Seoul 06273, Korea; jiwonflu@yuhs.ac (J.W.); bhjinccf@hanmail.net (B.-H.J.); alfo0103@naver.com (M.L.); spinepjy@yuhs.ac (J.-Y.P.); 2 Brain Korea 21 PLUS Project for Medical Science, Yonsei University, Seoul 03722, Korea; 3 Biomedical Research Institute, Biohedron Therapeutics Co., Ltd., Seoul 06230, Korea; 4 Biomedical Research Center, Gangnam Severance Hospital, Yonsei University College of Medicine, Seoul 06230, Korea; moonsir@yuhs.ac; 5 Department of Neurosurgery, International ST Mary’s Hospital, College of Medicine, Catholic Kwandong University, Incheon 22711, Korea; 6 Department of Neurosurgery, College of Medicine, Yonsei University Graduate School, Seoul 03722, Korea; 7 College of Physicians and Surgeons, Columbia University Vagelos, New York, NY 10032, USA; euniceyjl33@gmail.com

**Keywords:** transparent embryo, embryo clearing, passive clearing technique, IMPACT, PRDM10, PRDM13

## Abstract

Recent developments in tissue clearing methods have significantly advanced the three-dimensional analysis of biological structures in whole, intact tissue, providing a greater understanding of spatial relationships and biological circuits. Nonetheless, studies have reported issues with maintaining structural integrity and preventing tissue disintegration, limiting the wide application of these techniques to fragile tissues such as developing embryos. Here, we present an optimized passive tissue clearing technique (PACT)-based embryo clearing method, initial embedding PACT (IMPACT)-Basic, that improves tissue rigidity without compromising optical transparency. We also present IMPACT-Advance, which is specifically optimized for thin slices of mouse embryos past E13.5. We demonstrate proof-of-concept by investigating the expression of two relatively understudied PR domain (PRDM) proteins, PRDM10 and PRDM13, in intact cleared mouse embryos at various stages of development. We observed strong PRDM10 and PRDM13 expression in the developing nervous system and skeletal cartilage, suggesting a functional role for these proteins in these tissues throughout embryogenesis.

## 1. Introduction

Significant recent advancements in the field of tissue clearing have allowed for the appreciation of molecular patterns and cellular circuits in various biological tissues in three-dimensional space. As opposed to traditional immunohistochemistry in frozen or paraffin sections, clear lipid-exchanged acrylamide-hybridized rigid imaging/immunostaining/in situ-hybridization-compatible tissue-hydrogel (CLARITY)-based methods enable the microscopic study of tissue architecture in intact whole tissues and organs [1,2]. We recently reported the development of novel passive tissue clearing techniques (PACTs), process-separated PACT (psPACT) and modified PACTs (mPACT and mPACT-A), which significantly reduced required tissue processing times while improving achieved optical transparency [3,4,5].

A full understanding of the biological processes that shape a developing embryo requires the study of these phenomena across not only time but, when possible, also three-dimensional space [6,7]; the complexities of organ formation and development are not adequately captured in two-dimensional sections that are used in traditional immunohistochemistry. Processing developing embryos via CLARITY-based methods for this purpose, however, has proven challenging because the fragility of embryonic tissues leads to their disintegration upon exposure to the harsh treatments required by the majority of tissue clearing protocols. Though studies have sought to address this issue, currently published methods specific to clearing vertebrate embryos are limited by either long processing times or the use of organic solvents, which are known to produce artifacts in subsequent immunostaining [5].

Here, we present an optimized version of our previously published mPACT method that is specifically geared towards clearing mouse embryos [3,5]. The protocol, which we refer to as IMPACT-Basic (initial embedding PACT), maintains tissue integrity without compromising achieved optical transparency. We also present IMPACT-Advance, which allows for the clearance of thin sections of mouse embryos. IMPACT-Advance addresses the limitations imposed by the narrow 2-mm working distance of traditional confocal microscopes, which prevents the study of larger samples including mouse embryos past E13.5. To demonstrate proof-of-concept, we investigated the expression of PR domain 10 (PRDM10) and PRDM13, two members of the PRDM (PRDI-BF1 and RIZ homology domain-containing) family [8,9], in mouse embryos cleared via IMPACT-Basic and IMPACT-Advance. PRDMs have emerged as important transcriptional regulators that control the development of numerous organ systems throughout embryogenesis. While both PRDM10 and PRDM13 have been implicated in key developmental processes, such as cell fate specification, and in various cancer [10,11,12,13,14,15,16,17,18,19], they remain significantly less well-characterized compared to their counterparts in the PRDM family [8,9,20]. Our studies not only demonstrate the applicability and efficacy of the IMPACT methods for clearing embryonic tissue but also provide the first three-dimensional survey of PRDM10 and PRDM13 expression in the developing mouse embryo.

## 2. Results

### 2.1. Generation of Transparent Mouse Embryos Using a PACT-Based Modified Tissue Clearing Method

**Figure 1:**
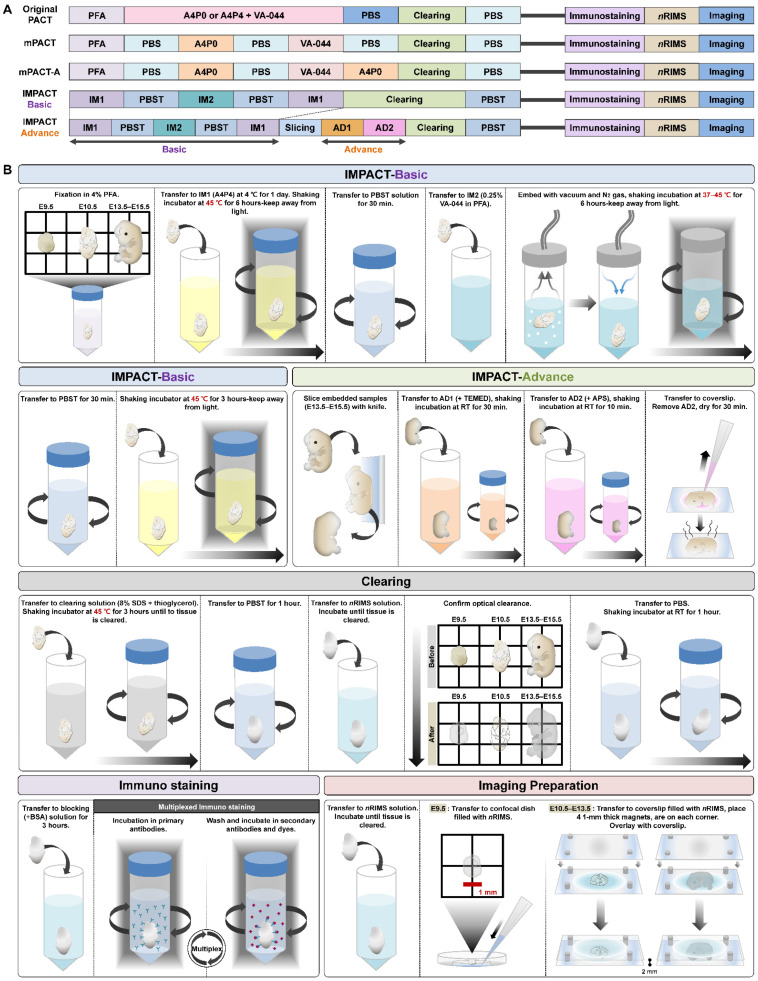
Schematic representation of passive tissue clearing methods. (**A**) The individual reagents or processes used for polymerization in the passive clearing methods are shown, including the additional incubation steps in polymerization solution (4% acrylamide and 4% paraformaldehyde (PFA) in phosphate-buffered saline (PBS) (IM1), 0.25% 2,2′-azobis[2-(2-imidazolin-2-yl)propane]dihydrochloride (VA-044) and 4% PFA in PBS (IM2), 4% acrylamide-based solution containing N,N,N′,N′-tetramethyl ethylenediamine (TEMED) (AD1), and 4% acrylamide-based solution containing ammonium persulfate (APS) (AD2)) in the initial embedding passive tissue clearing technique (IMPACT)-Basic and IMPACT-Advance protocols. (**B**) Schematic representation of IMPACT optimized for mouse embryos. The steps for IMPACT-Basic and IMPACT-Advance, are drawn in greater detail.

**Figure 2:**
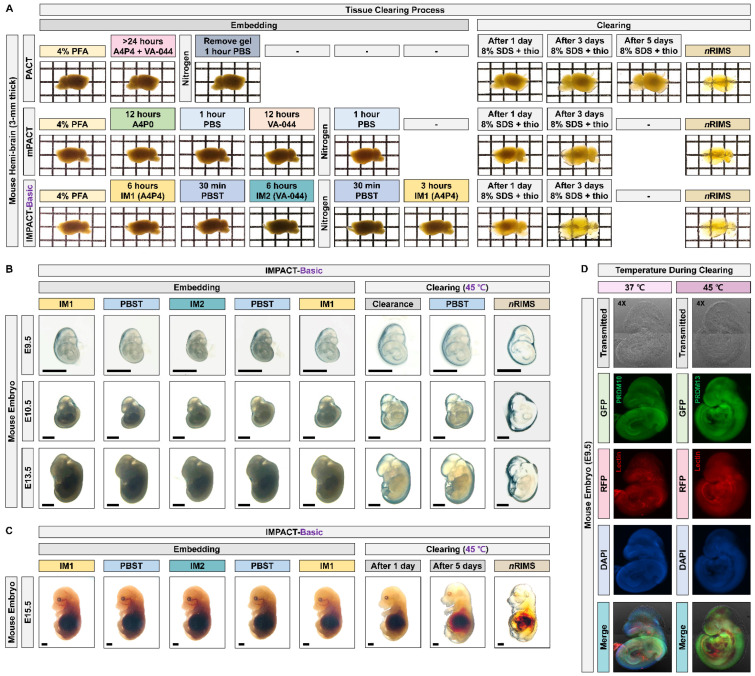
Generation of transparent mouse embryos via IMPACT-Basic. (**A**) Comparison of optical transparency in the mouse hemi-brain achieved by PACT, modified PACT (mPACT), and IMPACT-Basic. The transparency of all cleared samples was assessed against a patterned background (length:width = 5 mm:5 mm). (**B**,**C**) Comparison of optical transparency in E9.5, E10.5, E13.5, and E15.5 mouse embryos achieved by IMPACT-Basic (black scale bar: 2 mm). (**D**) Comparison of optical and fluorescence images in E9.5 mouse embryos achieved by clearing at 37 and 45 °C using IMPACT-Basic. The whole image of each sample was created using fluorescent microscopy, and the microscope was focused on 1 × 2 panels (horizontal × vertical). Merged images are with PR domain 10 (PRDM10) and PRDM13 in green, lectin in red, and DAPI in blue.

To investigate the expression of PRDM10 and PRDM13 in intact embryos and central nervous system (CNS) tissue, we sought to optimize our previously described mPACT and mPACT-A protocols to both accelerate clearing and improve the preservation of tissue integrity. In mPACT and mPACT-A, samples are fixed in paraformaldehyde (PFA), treated with A4P0 (4% acrylamide in phosphate-buffered saline (PBS)) and 0.25% 2,2′-azobis[2-(2-imidazolin-2-yl)propane]dihydrochloride (VA-044), and they are embedded with vacuum and nitrogen gas followed by either a PBS wash or A4P0, respectively, prior to clearing. Here, we fixed samples with IM1 (4% acrylamide and 4% PFA in PBS), followed by treatment with IM2 (0.25% VA-044 and 4% PFA in PBS). After embedding, samples were re-incubated in IM1 prior to clearing (Figure 1A). In the newly optimized mPACT (IMPACT-Basic) procedure, incubation and clearing was performed at 45 °C rather than at the 37 °C temperature of the standard procedure. This protocol, which we refer to as IMPACT-Basic, yielded firmer tissues post-clearance. Figure 1B provides a detailed outline of the steps required in IMPACT-Basic. Though previous reports suggested that the IM2 solution can decrease the efficacy of tissue clearing [2], mouse brain tissues processed via IMPACT-Basic achieved a higher level of optical transparency as PACT and mPACT within the same time frame. After demonstrating the feasibility of IMPACT-Basic, we then applied this protocol to developing mouse embryos at various stages, all of which were successfully cleared (Figure 2 and Figure S1A).

**Figure 3:**
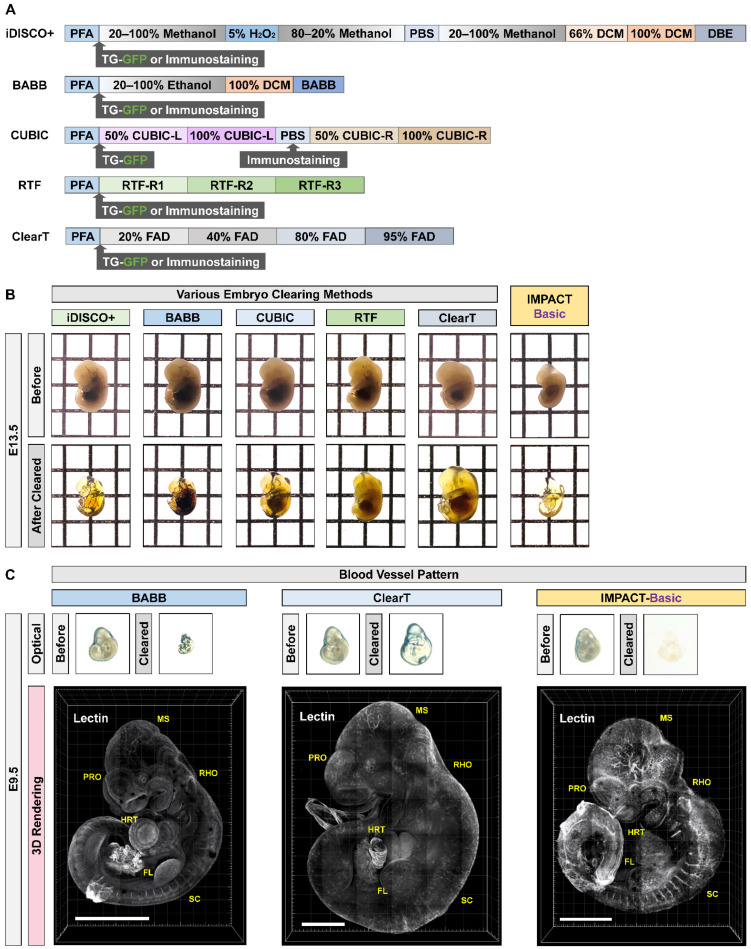
Generation of transparent mouse embryos via passive clearing methods. (**A**,**B**) Schematic representation of passive tissue clearing methods. The individual reagents or processes used for dehydration and clearing process in the four clearing methods are shown. Comparison of optical transparency achieved in E13.5 processed via iDISCO+, BABB, CUBIC, RTF, Clear*^T^* and IMPACT-Basic. The transparency of all cleared samples was assessed against a patterned background (length:width = 5 mm:5 mm). (**C**) Comparison of optical and lectin images in E9.5 processed via BABB, Clear*^T^*, and IMPACT-Basic. The whole image of each sample was created from serial z-images (25 slices) of the blood vessel pattern using confocal microscopy, and the microscope was focused on 3 × 4, 6 × 7, and 4 × 5 panels (horizontal × vertical). PRO = prosencephalon; MS = mesencephalon; RHO = rhombencephalon; SC = spinal cord; HRT = heart; FL = fore limb. White scale bar: 1000 µm.

To investigate the expression profiles of PRDM10 and PRDM13, we performed immunostaining for the two proteins (PRDM10 and PRDM13) at E9.5 after clearing alongside lectin staining to visualize blood vessels. Cleared mouse embryo of IMPACT-Basic methods showed blood vessels and PRDM expression while preserving proteins in at the E9.5 mouse embryo after clearing (Figure 2D and Figure S2). We also compared the reported embryo clearing protocols (iDISCO+, BABB, CUBIC, RTF and Clear*^T^*) and IMPACT-Basic in at E13.5 mouse embryos. IMPACT-Basic achieved the best clearance, as observed by eye (Figure 3A,B and Figure S3). We performed immunostaining for lectin to visualize a blood vessel in an E9.5 mouse embryo cleared via IMPACT-Basic and compared it to the blood vessel visualization in embryos cleared via BABB and Clear*^T^*. As a result, morphological structures of E9.5 mouse embryos processed with IMPACT-Basic were observed at higher resolutions than with BABB and Clear*^T^* (Figure 3C). These results demonstrated the use of IMPACT-Basic to visualize molecular patterns in whole mouse embryos and, for the first time, showed the three-dimensional expression of PRDM10 and PRDM13 in a developing mouse.

### 2.2. Profiling of PRDM10 and PRDM13 Expression in Early-Stage Mouse Embryos via IMPACT-Basic

**Figure 4:**
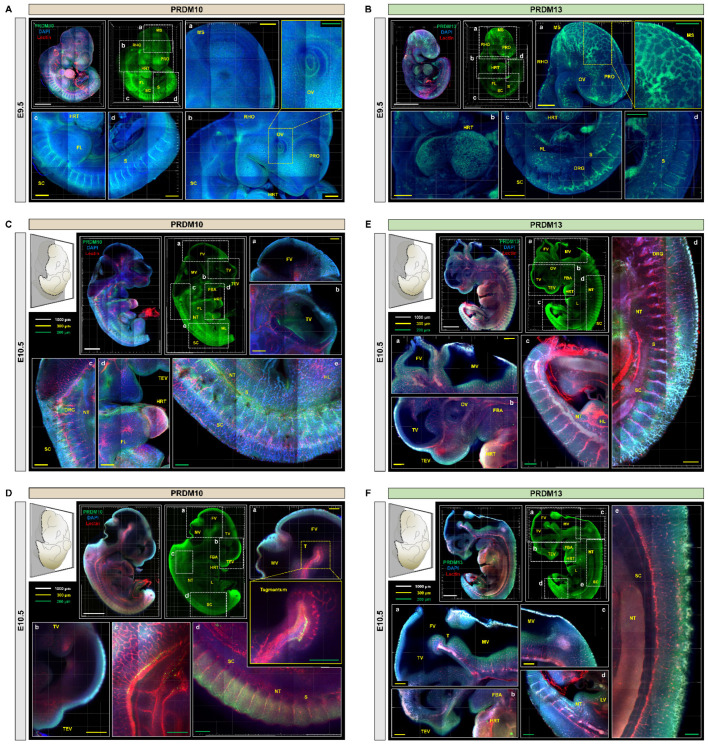
Profiling of PRDM10 and PRDM13 expression during mouse development in intact embryos processed via IMPACT. (**A**) PRDM10 and DAPI immunostaining in E9.5 mouse embryo processed via IMPACT-Basic. Zoom-in images of mesencephalon region (a), prosencephalon and rhombencephalon regions (b), and spinal cord region and developing somites (c,d). (**B**) PRDM13 and DAPI immunostaining in E9.5 mouse embryo. Zoom-in images of craniofacial and rhombencephalon regions (a), heart (b), and spinal cord region and developing somites (c,d). PRO = prosencephalon; MS = mesencephalon; RHO = rhombencephalon; SC = spinal cord; CNP = caudal neuropore; HRT = heart; OV = optic vesicle; S = somite pairs; FL = fore limb. (**C**) Sagittal sections of PRDM10 and lectin immunostaining at E10.5. Zoom-in images of midbrain (a), craniofacial region (b), dorsal region (c), ventral region (d), and spinal cord region (e). (**D**) Additional regions in which PRDM10 expression was observed at E10.5. Zoom-in images of midbrain (a), craniofacial region (b), dorsal region (c), and spinal cord region (d). (**E**) Sagittal sections of PRDM13 and lectin immunostaining at E10.5. Zoom-in images of midbrain (a), craniofacial region (b), tail region (c), and spinal cord region (d). (**F**) Additional regions in which PRDM13 expression was observed at E10.5. Zoom-in images of midbrain (a), craniofacial region (b), dorsal region (c), tail region (d), and spinal cord region (e). All images were tile scanned and z-stacked. DRG = dorsal root ganglion; FBA = first branchial arch; FV = fourth ventricle; HRT = heart; MV = mesencephalic vesicle; SC = spinal cord; NT = neural tube; TEV = telencephalic vesicle; TV = third ventricle; T = tegmentum; FL = fore limb; L = liver. Scale bars are as follows: white: 1000 µm; yellow: 300 µm; and green: 200 µm).

PRDM family proteins function as either direct histone methyltransferases or modulators of epigenetic regulators. Despite the pleiotropic roles of PRDM10 and PRDM13 in both development and pathological states such as cancer, little is known about their expression profiles. We therefore performed immunostaining for PRDM10 and PRDM13 in mouse embryos processed via IMPACT-Basic, which allowed us to survey their expression in a three-dimensional manner in whole, intact mouse embryos without being restricted to a single tissue or organ system. At E9.5, we observed high PRDM10 and PRDM13 expression in craniofacial structures and developing somites, as well as in the brain and the developing notochord (Figure 4A,B and Videos S1 and S2). Within the brain, both proteins were specifically expressed in the developing telencephalon, tegmentum, cerebellum, midbrain, dorsal root ganglia, and hindbrain. At E10.5, PRDM10 and PRDM13 expression was observed in developing craniofacial structures, CNS, somites, heart, and tegmentum (Figure 4C–F and Figure S4A). Especially strong expression was observed in the spinal cord, with segmental expression in the spinal ganglia. These results provided a proof-of-concept demonstration that our embryo-specific IMPACT-Basic method can be used to perform three-dimensional analyses of biological structures in whole intact embryos.

### 2.3. Development of Embryo-Specific IMPACT-Advance to Achieve Tissue Clarity and Retain Intact Organs in Large Embryo Section

**Figure 5:**
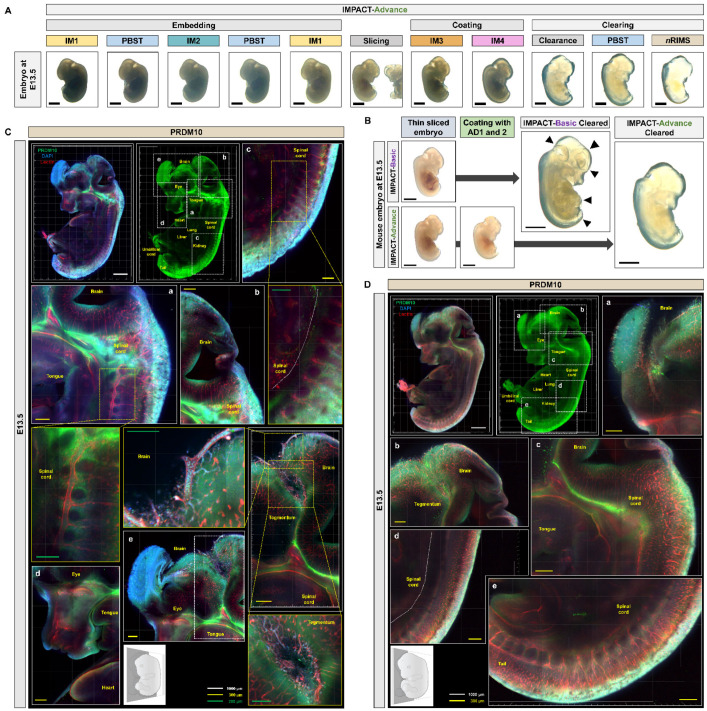
PRDM10 expression in mouse embryos at E13.5. (**A**) Comparison of optical transparency achieved in E13.5 mouse embryos via IMPACT-Advance. (**B**) Comparison of optical transparency achieved in E13.5 mouse embryos processed via IMPACT-Basic and IMPACT-Advance. Black arrows point to roughened surface of regions that experienced swelling after clearing via IMPACT-Basic, which were not observed in tissues processed by IMPACT-Advance. (**C**) PRDM10 and lectin immunostaining in E13.5 embryos processed via IMPACT-Advance. Zoom-in images of dorsal region (a), fourth ventricle (b), spinal cord region (c), craniofacial region (d), and midbrain (e). (**D**) Additional regions in which PRDM10 expression was observed at E13.5. Zoom-in images of craniofacial region (a), midbrain and fourth ventricle (b), dorsal region (c), and spinal cord and tail regions (d,e). All section images were tile scan and z-stacked (range: 130 µm). Scale bars are as follows: black: 2 mm; white: 1000 µm; yellow: 300 µm; and green: 200 µm.

While IMPACT-Basic successfully allowed for the imaging of embryos up to E10.5, we found that embryos at and after E13.5 were difficult to assess due to the limited 2-mm working distance of the objective lens in conventional confocal laser microscopy. Large samples can be visualized using light-sheet microscopy, but the technique is not amenable to most traditional laboratories due to limited equipment and high associated costs [21]. Previous studies have demonstrated the application of PACT-based methods on tissue sections as a workaround, but mouse embryo sections, which are highly fragile, disintegrate upon PACT treatment. To address this issue, we further optimized our IMPACT-Basic method to specifically process sections of mouse embryos at E13.5–E15.5, hereafter referred to as IMPACT-Advance. IMPACT-Advance follows the same steps as IMPACT-Basic, but after the second incubation in IM1, embryos are sliced into thin sections at a thickness of roughly a third of the original embryo and incubated in AD1 (4% acrylamide-based solution containing N,N,N′,N′-tetramethyl ethylenediamine (TEMED)) and AD2 (4% acrylamide-based solution containing ammonium persulfate (APS)) prior to clearing (Figure 1). IMPACT-Advance successfully achieved the optical clearance of E13.5 mouse embryo sections within 24 h with little tissue damage (Figure 5A,B, and Figure S1B).

### 2.4. Profiling of PRDM10 and PRDM13 Expression in E13.5 Mouse Embryos via IMPACT-Advance

**Figure 6:**
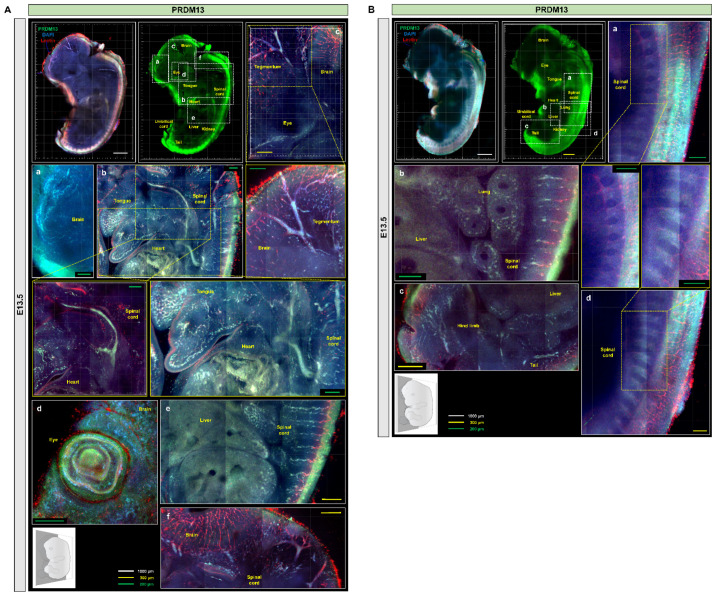
PRDM13 expression in mouse embryos at E13.5. (**A**) PRDM13 and lectin immunostaining at E13.5. Zoom-in images of craniofacial region (a), tongue and spinal cord regions (b), midbrain (c), eye (d), liver (e), and fourth ventricle and dorsal regions (f). (**B**) Additional regions in which PRDM13 expression was observed at E13.5. Zoom-in images of dorsal region (a), liver and lung regions (b), tail and hind limb regions (c), and spinal cord region (d). All section images were tile scan and z-stacked (range: 130 µm). Scale bars are as follows: white: 1000 µm; yellow: 300 µm; and green: 200 µm.

We then performed the immunostaining of PRDM10 and PRDM13 in E13.5 mouse embryo slices processed via IMPACT-Advance. At E13.5, PRDM10 expression was still concentrated in the developing brain, spinal cord, and skeletal cartilage, but it was also observed in the ventricle, tongue, olfactory epithelium, and umbilical cord (Figure 5C,D and Figure S4B, and Video S3). These results were in concordance with previous published studies on PRDM10 expression in mice [22]. Similar to PRDM10, PRDM13 was also expressed in the developing brain, spinal cord, and skeletal cartilage, but it was also observed in the lung, olfactory epithelium, and the eye (Figure 6A,B). The especially high levels of PRDM10 and PRDM13 expression in major blood vessels, the CNS, and skeletal cartilage suggest that these proteins may play critical functional roles in the development of these tissues.

## 3. Discussion

Here, we demonstrate the use of two novel tissue clearing protocols, IMPACT-Basic and IMPACT-Advance, specifically developed to process mouse embryos. While the original CLARITY method significantly advanced our understanding of three-dimensional relationships between biological structures with unprecedented detail, its relatively harsh treatments are not amenable to clearing mouse embryos. In response, various methodologies have been developed specifically for embryonic mouse tissue, but they are not without their own limitations [23]. For instance, Scale [24], Clear*^T^* [25], and SeeDB [26] require over a week to achieve optical transparency. In contrast, iDISCO+ [27], BABB [28], RTF [29], and CUBIC [21,30] rapidly generate transparent embryos, but their use of organic solvents can interfere with immunostaining and produce undesired artifacts [5]. Furthermore, their clearing protocols require immunostaining prior to clearing the tissue of interest (Figure 3 and Figure S2). We also compared the blood vessel visualization of E9.5 mouse embryos processed with BABB, Clear*^T^*, and IMPACT-Basic. The embryonic blood vessels were clearly observed in clear and high-resolution images after applying IMPACT-Basic.

Our IMPACT-Basic and IMPACT-Advance protocols are based on our previously described modified passive clearing techniques (mPACT and mPACT-A), which generate transparent tissues with high efficacy, limited equipment, and minimal hands-on processing time, as well as without the use of electrophoretic tissue clearing [3,4,5]. To improve tissue integrity, IMPACT-Basic requires sample fixation in IM1, which consists of 4% PFA and 4% acrylamide in PFA, followed by incubation in IM2 (0.25% VA-044 and 4% PFA in PBS). After embedding, samples are re-incubated in IM1 (Figure 1). Despite these modifications, IMPACT-Basic cleared processed tissues in the same time frame as the original PACT and mPACT protocols, and it achieved the same, if not higher, levels of transparency (Figure 2A). Furthermore, when applied to mouse embryos at E9.5–E15.5, IMPACT-Basic generated clear tissues that remained intact with a minimal loss of tissue integrity (Figure 2B,C).

Despite the success of IMPACT-Basic, due to the limited 2-mm working distance of objectives in most confocal microscopes, it is difficult to image embryos past E13.5. Furthermore, light-sheet microscopy, which allows for the imaging of larger samples, is not available in most laboratories for routine use. Therefore, we further optimized IMPACT-Basic for clearing thin sections as opposed to whole mouse embryos. This protocol, which we termed IMPACT-Advance, involves processing whole mouse embryos via IMPACT-Basic up to the re-incubation step in IM1, followed by thin-slicing and subsequent incubation in AD1 (4% acrylamide-based solution containing TEMED) and AD2 (4% acrylamide-based solution containing APS) (Figure 1). Slices of E13.5 embryos were successfully cleared via IMPACT-Advance with minimal damage to tissue integrity (Figure 5A,B).

To demonstrate proof-of-concept, we investigated the expression of two PRDM family proteins, PRDM10 and PRDM13, in mouse embryos cleared via IMPACT-Basic and IMPACT-Advance methods (Figure 4, Figure 5 and Figure 6). PRDM10 and PRDM13 have been implicated in both vertebrate development and cancer, but their expression profiles and function are less well-characterized relative to other members of the PRDM family. Probing their expression in an intact embryo allowed for a broad survey of their expression profiles in the entire embryo without being restricted to any one organ system or tissue type; furthermore, it allowed for an appreciation of three-dimensional relationships between PRDM-expressing tissues that could potentially uncover novel findings about their functions. Consistent with previous reports of PRDM10 and its role in the development of sensory neurons, we showed that PRDM10 is expressed in the developing nervous system at E9.5, E10.5, and E13.5, including the neural crest, olfactory epithelium, notochord, and dorsal root ganglia, as well as other regions in the developing brain and spine. We also observed PRDM10 expression in craniofacial structures and cartilage formation. Similarly, we observed PRDM13 expression primarily in craniofacial structures and CNS tissues such as the notochord, eye, and olfactory epithelium, with an especially high expression in the spinal ganglia. Additional areas of PRDM13 expression included the lung and the adrenal medulla. Both proteins were expressed in a segmental fashion in somites, spinal cord, heart, and tegmentum at E10.5 and E13.5 (Table S2).

Our results established, for the first time, the expression profiles of PRDM10 and PRDM13 in intact whole mouse embryos at various developmental stages. Consistent with the overexpression of PRDM10 and PRDM13 in numerous cancer types, both proteins were expressed in a wide spectrum of tissues in the developing embryo, supporting the need for future studies to probe their functional roles beyond what has been previously reported in the literature. Importantly, these studies have demonstrated the feasibility and efficacy of IMPACT-Basic and IMPACT-Advance for clearing tissues derived from mouse embryos, further broadening the applicability of CLARITY-based methods for studying biological structures.

## 4. Materials and Methods

### 4.1. Animal

Adult male and female ICR (Institute of Cancer Research) mice were purchased from Orient Inc. (Gyeonggi-do, Korea) and were raised in a specific pathogen-free (SPF) environment. Mouse embryos were isolated from E9.5 to E13.5. All experimental procedures were carried out in strict accordance with the recommendations provided by the Ministry of Agriculture, Food, and Rural Affairs (MAFRA) and were approved by the Institutional Animal Care and Use Committee (IACUC) at Yonsei University (licenses #2017-0230, Date of Approval: 10 March 2020).

### 4.2. Isolation of Mouse Embryo and Tissue

Upon opening the mouse thorax, an incision was made to the right atrium of the heart. Mice were then perfused with equal volumes of cold 0.1 M PBS with 10 unit/mL heparin (Sigma-Aldrich Inc., St. Louis, MO, USA) and 4% PFA (Biosesang Inc., Gyeonggi-do, Korea). Mouse embryos were then isolated using previously described methods. The sample was submerged in 4% PFA and stored at 4 °C for 24 h.

### 4.3. Original PACT and mPACT

For the original PACT protocol, samples were fixed in a fresh hydrogel monomer solution (A4P4; 4% acrylamide (Sigma-Aldrich Inc., St. Louis, MO, USA) and 4% PFA in 0.1 M PBS) containing 0.25% photoinitiator VA-044 (Wako Chemicals USA, Inc., Richmond, VA, USA) and stored 4 °C for 24 h. For mPACT, the sample was submerged in 4% PFA and stored at 4 °C for 24 h. Samples were washed with 0.1 M PBS and then submerged in A4P0 solution (4% acrylamide in 0.1 M PBS) at 37 °C for 24 h, followed by incubation in 0.25% VA-044 in 0.1 M PBS at 37 °C for 6 h. Samples were embedded with vacuum and nitrogen gas, each for 10 min. Tissues processed via the original PACT protocol were removed from the embedded hydrogel and transferred to clearing solution (8% sodium dodecyl sulfate (SDS; Affymetrix Inc., OH, USA) in 0.1 M PBS, pH 8.0) with 0.5% α-thioglycerol (Sigma-Aldrich Inc., St. Louis, MO, USA) in a shaking incubator at 37 °C and 150 rpm until optical transparency was achieved.

### 4.4. IMPACT-Basic and IMPACT-Advance

For IMPACT-Basic, samples were fixed in IM1 solution (A4P4: 4% acrylamide and 4% PFA in 0.1 M PBS) at 4 °C for 24 h, and then they were incubated in fresh IM1 solution at 37 °C for 6 h. After a 30 min wash in PBST (0.1% Triton X-100 (Sigma-Aldrich Inc., St. Louis, MO, USA) in 0.1 M PBS), samples were submerged in IM2 solution (0.25% VA-044 and 4% PFA in 0.1 M PBS). Samples were embedded with vacuum and nitrogen gas for 10 min, and then they were incubated at 45 °C for 6 h. Samples were then washed in PBST for 30 min and resubmerged in IM1 solution at 45 °C for 3 h.

For IMPACT-Advance, samples were processed via the IMPACT-Basic protocol until embedding, after which they were sagittally sliced with a knife under a stereoscopic microscope (SMZ745T; Nikon, Tokyo, Japan). Slices were placed in a shaking incubator at room temperature with 10 mL of AD1 (4% acrylamide, 0.1% bis-acrylamide (Sigma-Aldrich Inc., MO, USA), 4% PFA, and 1.3% TEMED; Amresco Inc., Solon, PA, USA) in 0.1 M PBS) for 30 min and then submerged in AD2 (4% acrylamide, 0.1% bis-acrylamide, 4% PFA, and 2% APS; Amresco Inc., Solon, PA, USA) in 0.1 M PBS) solution at room temperature for 10 min. Samples were transferred to 24 × 60-mm coverslips (Paul Marienfeld GmbH & Co., Lauda-Königshofen, Germany) and embedded at room temperature for 20 min, followed by incubation in clearing solution (8% SDS in 0.1 M PBS at pH 8.0) with α-thioglycerol (0.25%: E9.5 mouse embryo; 0.5%: E10.5–E15.5 mouse embryos and brain) at 45 °C and 150 rpm until optical transparency was achieved. For more detailed instructions, see Figure 1B.

### 4.5. RTF

After fixation in 4% PFA, samples were incubated in RTF-R1 solution (30% triethanolamine (Daejung Chemicals & Metals, Gyeonggi-do, Korea) and 40% formamide (Georgiachem, Norcross, GA, USA) in dH_2_O) for 6 h at room temperature, followed by incubation in RTF-R2 solution (60% triethanolamine and 25% formamide in dH_2_O) for 6 h at room temperature. Samples were then immersed in RTF-R3 solution (70% triethanolamine and 15% formamide in dH_2_O) at room temperature until they achieved optical clearance.

### 4.6. CUBIC

After fixation in 4% PFA, samples were immersed in 50% CUBIC-L (Tokyo Chemical Industry Co., Ltd., Tokyo, Japan) with 0.1 M PBS and incubated in 100% CUBIC-L solution at 37 °C for 72 h in a shaking incubator. Samples were washed in PBS for 24 h and pre-treated in 50% CUBIC-R (Tokyo Chemical Industry Co., Ltd., Tokyo, Japan) solution with 0.1 M PBS at room temperature for 24 h. Samples were then incubated in 100% CUBIC-R solution at room temperature for 48 h.

### 4.7. BABB

Embryos were fixed in 4% PFA at 4 °C for 24 h and then washed with 0.1 M PBS for 1 h before being hydrated to 20%, 50%, 80%, and 100% ethanol (Millipore Co., MA, USA) at 37 °C for 1 h each. Embryos were further washed with 100% ethanol at 4 °C for 1 h. Embryos were incubated in 100% dichloromethane (DCM; Sigma-Aldrich, Inc., St. Louis, MO, USA) at room temperature for 30 min, and the sample was washed with ethanol. The embryo was then incubated in BABB solution (1 volume of benzyl alcohol (BA; Sigma-Aldrich, Inc., St. Louis, MO, USA) to 2 volumes of benzyl benzoate (BB; Sigma-Aldrich, Inc., St. Louis, MO, USA)) at room temperature until the tissue cleared.

### 4.8. iDISCO+

Embryos were fixed in 4% PFA at 4 °C for 24 h and then washed with 0.1 M PBS for 1 h before being hydrated to 20%, 50%, 80%, and 100% methanol (Millipore Co., Burlington, MA, USA) at room temperature for 1 h each. Embryos were further washed with 100% methanol at 4 °C for 1 h. Embryos were incubated in 5% hydrogen peroxide H_2_O_2_ (1 volume of 30% H_2_O_2_ to 5 volumes of methanol) at 4 °C for 12 h. After treatment with 5% H2O2, the embryos were rehydrated to 80%, 60%, 40%, and 20% methanol with 0.1 M PBS at room temperature for 1 h each. After PBS washing, the embryos were dehydrated with 20%, 40%, 60%, and 100% methanol at room temperature for 1 h each; then, they were transferred to a glass bottle containing mixture of 66% DCM and 33% methanol, and they were incubated at room temperature for 3 h. Embryos were incubated in 100% DCM for 15 min, and each sample was washed with methanol. Each embryo was then incubated in dibenzyl ether (DBE; Sigma-Aldrich Inc., St. Louis, MO, USA) at room temperature until the tissue cleared.

### 4.9. Clear^T^

Following 4% PFA fixation, embryos were incubated in 20% formamide and 40% formamide (*vol/vol*) in 0.1 M PBS (pH 7.4) for 30 min each, followed by 80% formamide (*vol/vol*) and 95% formamide (*vol/vol*) for 2 h until they achieved optical clearance.

### 4.10. Immunostaining

Cleared embryos were incubated in PBST for 2 h and blocked with 2% bovine serum albumin (BSA; Sigma-Aldrich, Inc., St. Louis, MO, USA) in PBST for 6 h. Embryos were incubated with either anti-PRDM10 or anti-PRDM13 primary antibodies for 24–72 h, followed by 3 washes in PBST for 24–48 h each. They were then incubated in secondary antibody (goat anti-rabbit IgG H&L, Alexa Fluor^®^ 488), lectin dye (DyLight 594-labeled *Lycopersicon Esculentum* (Tomato) lectin), and DAPI in PBST for 24–72 h. Detailed information about antibodies and dyes used in this study is provided in Table S1.

*n*RIMS was prepared by mixing 0.8 g/mL Nycodenz (Axis-Shield Density Gradient Media, Oslo, Norway) in 30 mL of a base buffer (0.01% sodium azide (Sigma-Aldrich, Inc., St. Louis, MO, USA) and 0.1% Tween-20 in 0.1 M PBS; pH 7.5). Labeled embryos were washed three times with PBST for 24–72 h and stored in 5 mL of *n*RIMS solution for 6–24 h. The embryos at E9.5 were incubated in a small confocal dish (SPL Life Sciences Co., Gyeonggi-do, Korea) containing *n*RIMS and covered with a 24-mm coverslip (Paul Marienfeld GmbH & Co., Lauda-Königshofen, Germany). The embryos at E10.5–13.5 were covered in *n*RIMS and sandwiched between two 24 × 60-mm coverslips with small 1-mm thick magnets.

### 4.11. Image Processing

All clear images were captured using a digital camera (iPhone-X; Apple Inc., Elk Grove, CA, USA) and a stereoscopic microscope (SMZ745T; Nikon, Tokyo, Japan). Fluorescent microscopy was performed with an EVOS FL Cell Imaging System (Thermo Fisher Scientific, Waltham, MA, USA) at 4× magnification. Confocal microscopy was performed with an LSM-780 confocal microscope (Carl Zeiss, Oberkochen, Germany) at 10× magnification using the associated Zeiss software. Three-dimensional images and videos were edited into serial images using Imaris v8.01 software (Bitplane, Belfast, United Kingdom).

## Data Availability

The data presented in this study are available on request from the corresponding author.

## Supplementary Materials

The following are available online at https://www.mdpi.com/1422-0067/22/6/2892/s1. Figure S1: Comparison of optical transparency achieved in mouse embryos processed via IMPACT-Basic and IMPACT-Advance; Figure S2. Fluorescence images in E9.5 mouse embryos via IMPACT-Basic; Figure S3: Comparison of optical transparency in E13.5 mouse embryos achieved by iDISCO+, BABB, CUBIC, RTF, and Clear*^T^*; Figure S4: Blood vessel imaging in mouse embryos at E10.5 and E13.5; Table S1: Antibodies and dyes used in this study; Table S2: An outline of PRDM10 and PRDM13 mouse embryonic expression; Video S1: Three-dimensional images of PRDM10 immunostaining in a cleared E9.5 mouse embryo; Video S2: Three-dimensional images of PRDM13 immunostaining in a cleared E9.5 mouse embryo; Video S3: Three-dimensional images of PRDM10 immunostaining in focused midbrain in a cleared E13.5 mouse embryo.

## Abbreviations

PACT	Passive clearing technique
mPACT	Modified passive clearing technique
IMPACT	Initial embedding passive clearing technique
CNS	Central nervous system
PRDM	PR domain
CLARITY	Clear lipid-exchanged acrylamide-hybridized rigid imaging/immunostaining/in situ-hybridization-compatible tissue-hydrogel
