# Supplementary material for: Investigation of PRDM10 and PRDM13 Expression in Developing Mouse Embryos by an Optimized PACT-Based Embryo Clearing Method
Source: Int J Mol Sci. 2021 Mar 12;22(6):2892. doi: 10.3390/ijms22062892 (PMC8000312; doi:10.3390/ijms22062892)
Supplement: Supplementary file 1 [file ijms-22-02892-s001.zip › Supplementary Information.pdf]

Table S1. Antibodies and dyes used in this study.

	Antibody and dye	Marking target	Company	Cat No.	Ratio
**1**	PRDM10 rabbit antibody	Prdm10	Biorbyt	orb312613	1:100
**2**	PRDM13 rabbit antibody	Prdm13	Biorbyt	orb312614	1:100
**3**	Goat anti-rabbit IgG H&L (Alexa Fluor® 488)		Abcam	ab150077	1:100
**4**	DyLight 594 labeled Lycopersicon Esculentum (Tomato) Lectin (LEL, TL)	Blood vessel	Vector laboratories	DL-1177	-
**5**	DAPI Hoechst 33342	Nuclear	Invitrogen	R37605	-

Table S2. An outline of PRDM10 and PRDM13 mouse embryonic expression.

		PRDM10	PRDM13
**Mouse embryonic date**	**E9.5**	Craniofacial structures<br>Somites<br>Notochord<br>Telencephalon<br>Tegmentum<br>Cerebellum<br>Midbrain<br>Dorsal root ganglia<br>Hindbrain	Craniofacial structures<br>Somites<br>Notochord<br>Telencephalon<br>Tegmentum<br>Cerebellum<br>Midbrain<br>Dorsal root ganglia<br>Hindbrain
	**E10.5**	Craniofacial structures<br>Brain<br>Somites<br>Heart<br>Tegmentum<br>Spinal cord<br>Spinal ganglia	Craniofacial structures<br>Brain<br>Somites<br>Heart<br>Tegmentum<br>Spinal cord<br>Spinal ganglia
	**E13.5**	Brain<br>Spinal cord<br>Skeletal cartilage<br>Ventricle<br>Tongue<br>Olfactory epithelium<br>Umbilical cord	Brain<br>Spinal cord<br>Skeletal cartilage<br>Lung<br>Olfactory epithelium<br>Eye

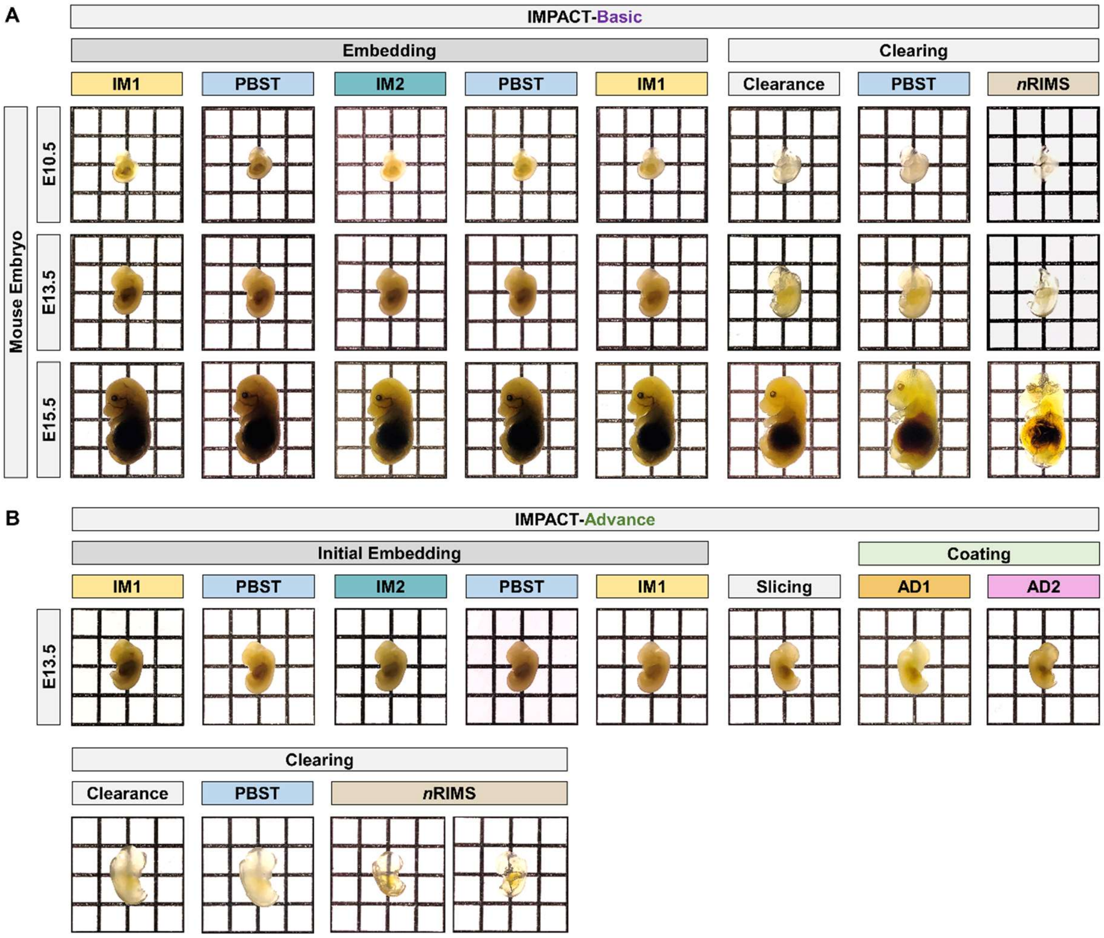

Figure S1. Comparison of optical transparency achieved in mouse embryos processed via IMPACT-Basic and IMPACT-Advance.

Comparison of optical transparency achieved in E9.5, E10.5, E13.5 and E15.5 mouse processed via IMPACT-Basic (**A**) and IMPACT-Advance (**B**). The transparency of all cleared samples was assessed against a patterned background (length:width=5-mm:5-mm).

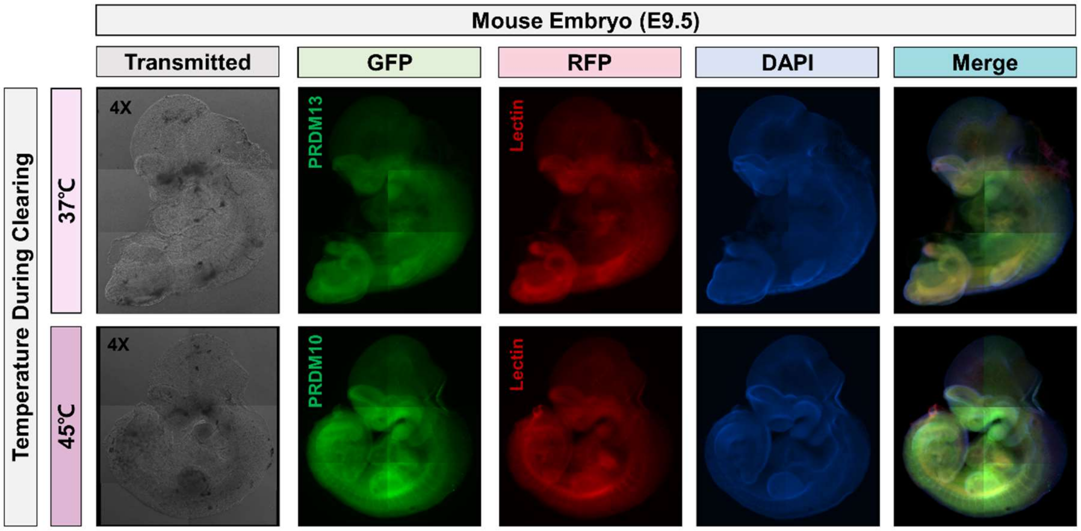

Figure S2. Fluorescence images in E9.5 mouse embryos via IMPACT-Basic.
Comparison of additional fluorescence images in E9.5 mouse embryos achieved by clearing at 37°C and 45°C using IMPACT-Basic. The whole image of each sample was created using fluorescent microscopy, and the microscope was focused on 2 × 3 panels (horizontal × vertical). Merged images with PRDM10 and PRDM13 in green, lectin in red, and DAPI in blue.

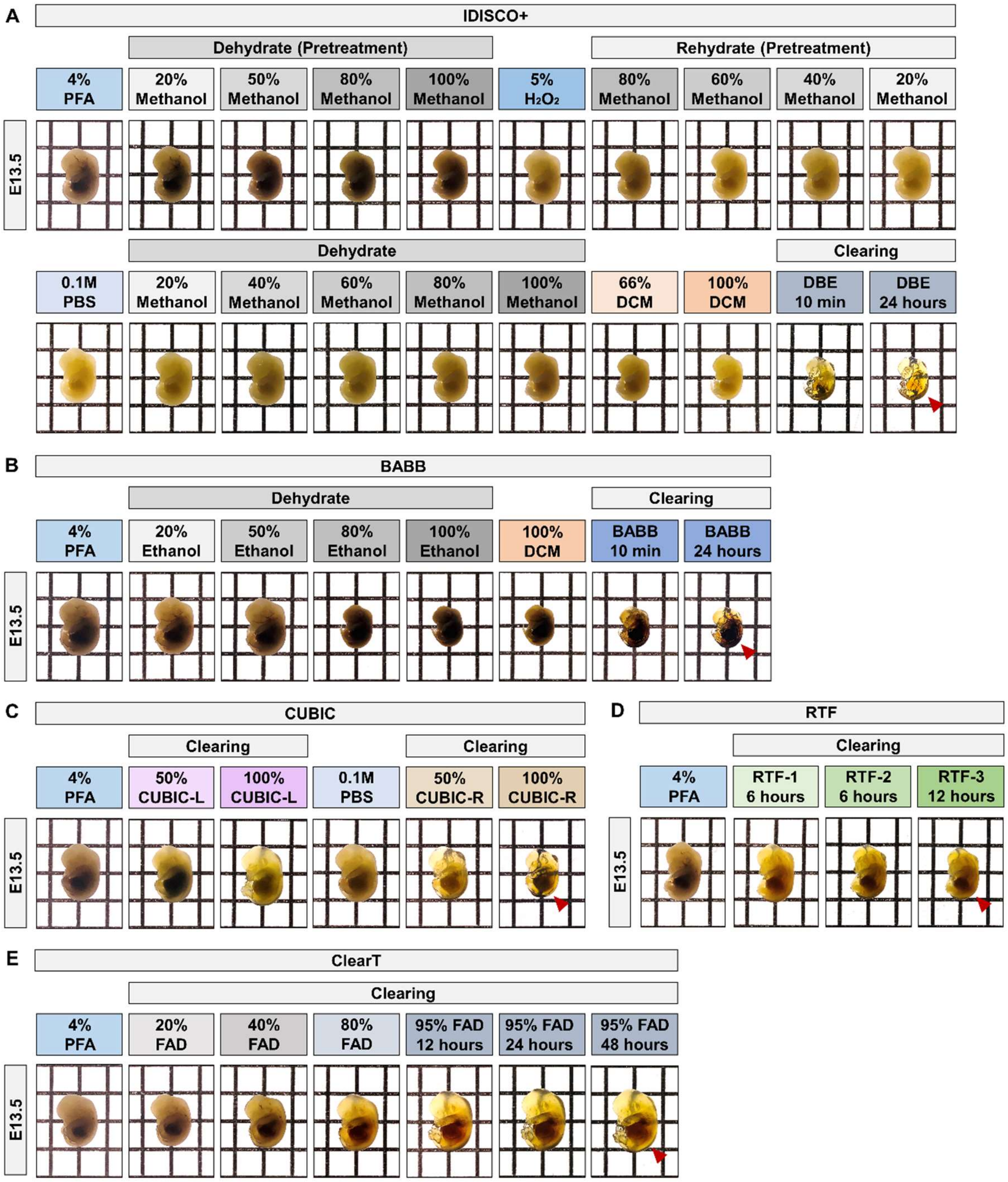

Figure S3. Comparison of optical transparency in E13.5 mouse embryos achieved by iDISCO+, BABB, CUBIC, RTF and Clear^T.

Comparison of optical transparency achieved in E13.5 processed via iDISCO+ (**A**), BABB (**B**), CUBIC (**C**), RTF (**D**) and Clear^T (**E**). The transparency of all cleared samples was assessed against a patterned background (length:width=5-mm:5-mm). Red arrows point to areas of opaque heart and liver.

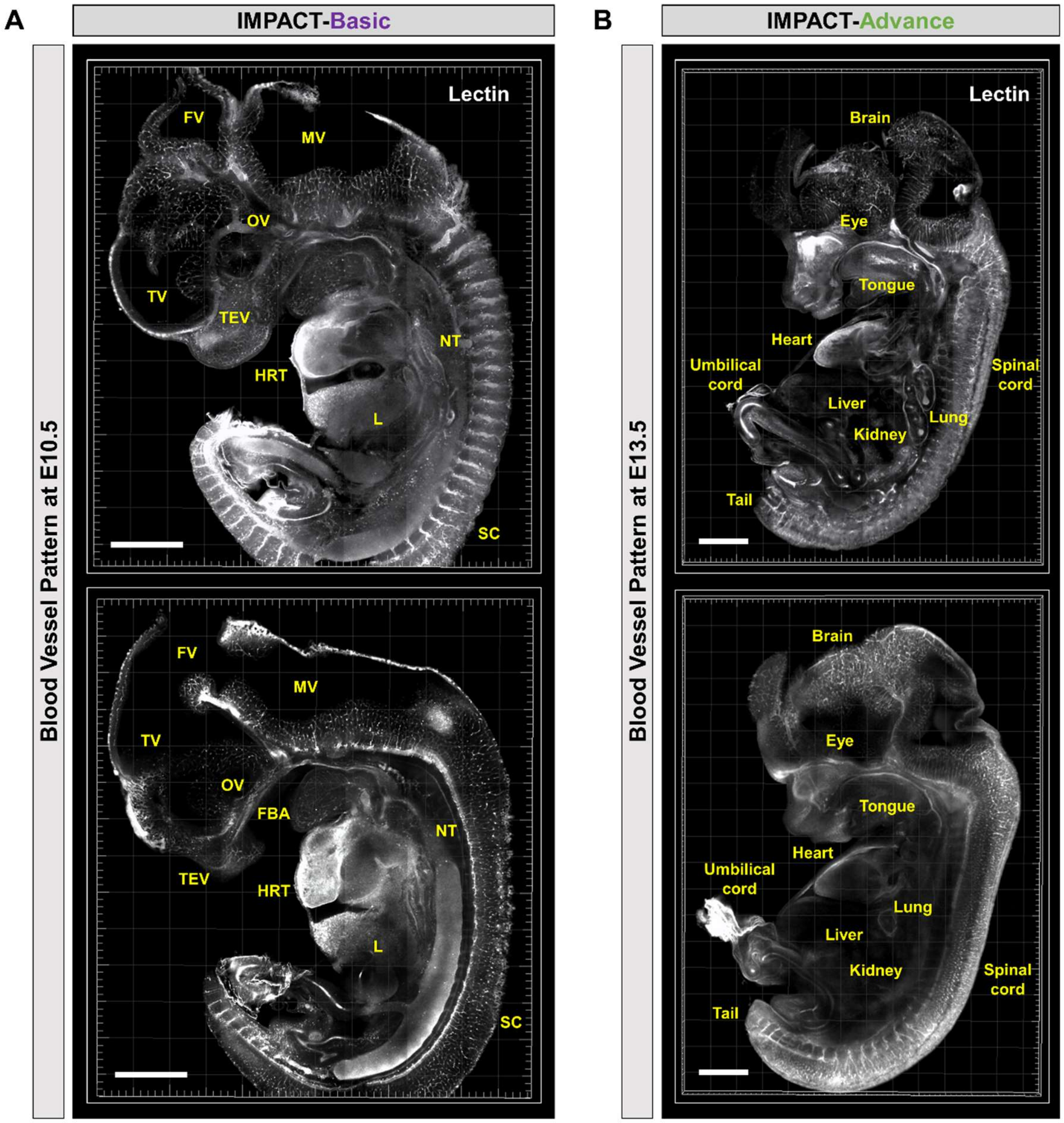

Figure S4. Blood vessel imaging in mouse embryos at E10.5 and E13.5.
Lectin immunostaining in E10.5 (**A**) and E13.5 (**B**) mouse embryos processed via IMPACT-Basic and IMPACT-Advance. FBA = first branchial arch; FV = fourth ventricle; HRT = heart; MV = mesencephalic vesicle; SC = spinal cord; NT = neural tube; TEV = telencephalic vesicle; TV = third ventricle; T = tegmentum; FL = fore limb; L = liver. Scale bar (white: 1000-µm).

Video S1. Three-dimensional images of PRDM10 immunostaining in a cleared E9.5 mouse embryo.

Video S2. Three-dimensional images of PRDM13 immunostaining in a cleared E9.5 mouse embryo.

Video S3. Three-dimensional images of PRDM10 immunostaining in focused midbrain in a cleared E13.5 mouse embryo.
